# Severe onset type 1 diabetes mellitus with unusual manifestations due to CD70 deficiency: novel pathway in deciphering the etiopathogenesis of type 1 diabetes mellitus, and the experience with hematopoietic stem cell transplantation

**DOI:** 10.48130/pedi-0026-0004

**Published:** 2026-06-22

**Authors:** Yagmur Unsal, Ertugrul Sancak, N. Berna Çelik Ertaş, David B. Langley, Hacer Neslihan Bildik, Aysegül Akarsu, Sevil Oskay Halacli, Dogus Vuralli Karaoglan, Saliha Esenboga, Diclehan Orhan, Baris Kuskonmaz, F. Visal Okur, E. Nazlı Gönç, Deniz Cagdas, Zeynep Alev Ozon

**Affiliations:** 1 Department of Pediatrics, Division of Pediatric Endocrinology, Hacettepe University Faculty of Medicine, Ankara 06100, Turkey; 2 Department of Pediatrics, Hacettepe University Faculty of Medicine, Ankara 06100, Turkey; 3 Immune Biotherapies Program, Garvan Institute of Medical Research, Sydney, Australia; 4 Institute of Child Health, Immunology, Hacettepe University Faculty of Medicine, Ankara 06100, Turkey; 5 Pediatric Immunology Laboratory, Hacettepe University Faculty of Medicine, Ankara 06100, Turkey; 6 Department of Pediatrics, Division of Pediatric Immunology, Hacettepe University Faculty of Medicine, Ankara 06100, Turkey; 7 Department of Pediatrics, Division of Pediatric Pathology, Hacettepe University Faculty of Medicine, Ankara 06100, Turkey; 8 Department of Pediatrics, Division of Pediatric Hematology, Hacettepe University Faculty of Medicine, Ankara 06100, Turkey

**Keywords:** Type 1 diabetes mellitus, CD70 deficiency, Inborn errors of immunity, Autoimmune, Hematopoietic stem cell transplantation

## Abstract

CD70 deficiency, mostly recognized by immune dysregulation, hemophagocytic lymphohistiocytosis, lymphoproliferation, and malignancy, was recently associated with severe and accelerated diabetes in mice. Such a causal relationship is not previously reported in humans. The aim of this study is to describe the first case of CD70 deficiency manifesting with severe onset type 1 diabetes mellitus (T1DM). Changes to glycemic control following hematopoietic stem cell transplantation and accompanying autoimmunity are defined. Immunophenotype analysis, next-generation-primary immunodeficiency panel analysis, and Sanger sequencing were conducted. Then, segregation analysis and functional studies were carried out for validation of the homozygous missense variant in *CD70*. Following confirmation of CD70 deficiency, hematopoietic stem cell transplantation (HSCT) was carried out. A comprehensive review of clinical history, alongside biochemical and immunologic investigations, was carried out to identify the natural history as well as immune and endocrine phenotypes. Presentation of severe-onset T1DM with severe diabetic ketoacidosis, multiorgan dysfunction, and unexplained findings during follow-up (recurrent diabetic ketoacidosis episodes, unexplained recurring skin lesions, and celiac disease) prompted an evaluation for underlying etiology, and CD70 deficiency was diagnosed. Timely HSCT was curative for CD70 deficiency. Following HSCT, glycemic control was improved (median HbA1c: 8.1% [7.6–8.6] vs 6.5% [5.8–7.0]), total daily insulin dose was reduced from 0.85 to 0.62 U/kg/d, and recurrent episodes of diabetic ketoacidosis ceased. It was concluded that, a homozygous missense variant in CD70, causing CD70 deficiency, may be associated with severe onset T1DM, confirming the significance of intact CD27/CD70 co-stimulation in the prevention of pancreatic *β*-cell autoimmunity. The CD27/CD70 pathway represents a potential target for immunotherapy trials in T1DM.

## Introduction

Inborn errors of immunity (IEIs) result in increased susceptibility to infections, but their manifestations extend beyond. Defects in immune tolerance, apoptosis, and autophagy lead to a variety of clinical manifestations, including autoimmunity, autoinflammation, allergy, asthma, and malignancy, all stemming from immune dysregulation^[1]^. Autoimmunity can be organ- and tissue-specific, with endocrine organs as primary targets, and thyroid autoimmunity being the most common^[2]^.

Type 1 diabetes mellitus (T1DM), the autoimmune disease characterized by progressive destruction of pancreatic *β*-cells, leading to absolute insulin deficiency and disruption of glucose metabolism^[3]^, is multifactorial, involving both monogenic and polygenic factors, as well as environmental influences. Genome-wide association studies have identified 143 regions comprising nearly 60 independent candidate genes involved in T-cell activation, signaling, antigen recognition, and tolerance associated with susceptibility to T1DM^[4]^. IEIs resulting in immune dysregulation that are associated with higher T1DM prevalence are IPEX syndrome (immune dysregulation, polyendocrinopathy, enteropathy, X-linked; 77%–95%), pathogenic variants of STATs (signal transducers and activators of transcription, ie. STAT1 gain-of function (GOF), STAT3 GOF; 23.8%, and STAT5b GOF), APECED syndrome (autoimmune polyendocrinopathy, candidiasis, ectodermal dystrophy; 1%–18%), activated phosphoinositide 3-kinase delta syndrome, cytotoxic T-lymphocyte–associated protein 4 haploinsufficiency, lipopolysaccaride-resposive and beige-like anchor protein deficiency^[2,5−7]^. Understanding the pathophysiologic mechanisms in specific IEIs is crucial as it may offer significant insights for understanding the pathophysiology, prevention, and treatment of T1DM in the general population.

Biallelic mutations of genes encoding CD27 or its ligand CD70 are known to result in IEIs manifesting predominantly as Epstein-Barr virus (EBV)-associated immune dysregulation, hemophagocytic lymphohistiocytosis, lymphoproliferation, and malignancy. Autoinflammatory symptoms such as periodic fever, aphthous stomatitis, pharyngitis, and cervical adenitis have been identified years prior to the initial disease manifestations of CD27 or CD70 deficiency^[8]^. Lately, *CD70* double-knockout (DKO) non-obese diabetogenic (NOD) mice were shown to develop more severe and accelerated diabetes compared to wild-type NOD^[9]^. CD70 was interpreted to elicit immune effects that restrict the development of T1DM. The association between CD70 deficiency and T1DM has not been previously documented in human subjects.

This report describes the first case of CD70 deficiency manifesting with severe onset T1DM with unusual manifestations. Immunologic evaluation was deemed necessary due to the severe onset T1DM and unexplained additional findings during follow-up. Results of hematopoietic stem cell transplantation (HSCT) on glycemic regulation are also being presented. This case significantly contributes to the understanding of T1DM susceptibility in specific IEIs, offering valuable insights for the prevention and treatment of T1DM in the general population.

## Materials and methods

This is a retrospective study carried out in a tertiary center. Medical history, presenting symptoms, age at diagnosis, family history, auxologic measurements (bodyweight [BW], height) and findings on physical examination, endocrine evaluations (blood glucose, insulin, c-peptide, HbA1c, autoantibodies to glutamic acid decarboxylase [anti-GAD_65_] and tyrosine phosphatase islet antigen 2 [anti-IA-2], ACTH, cortisol, thyroid stimulating hormone [TSH], free thyroxine [FT4]), liver and renal function tests, were recorded on admission and on the last clinical visit. T1DM was diagnosed using the American Diabetes Association Diagnostic Criteria^[10]^. Auxologic and endocrine evaluations (blood glucose, HbA1c, urinary ketone) were carried out quarterly as part of routine follow-up, and when clinically indicated. Chronic complications of T1DM (i.e., nephropathy, retinopathy) were screened yearly.

Auxologic measurements were made by a nurse trained in auxology. BW was measured as kilograms (kg) using a digital weighing scale (SECA 769, Hammer Steindamm, 22089, Hamburg, Germany), wearing lightweight clothing with a sensitivity of 0.1 kg. Height was measured in meters (m) in the standing position using a stadiometer (SECA 264, Hammer Steindamm, 22089, Hamburg, Germany) with a sensitivity of 0.1 cm. BW and height standard deviation scores (SDS) for chronologic age were calculated using gender specific charts of the Centers for Disease Control (CDC).

Autoantibodies to anti-GAD_65_ and anti-IA-2 were measured using commercial kits (DIAsource Anti-GAD_65_ RIA Kit, DIAsource Immunoassays S.A. B1348 Louvain-la-Neuve, Belgium, and DIAsource Anti-IA_2_ RIA Kit, DIAsource Immunoassays S.A. B1348 Louvain-la-Neuve, Belgium, respectively), which use a direct assay based on the principle of radioligand assays. For anti-GAD_65_, the upper normal limit is < 1 U/ml, intra- and inter-assay coefficient of variations are 10% and 20%, respectively, and sensitivity is 0.7 U/ml. The upper normal limit for anti-IA-2 is < 1 U/ml while the coefficient of variation is 10% within assay and 20% between assays, and the sensitivity is 0.8 U/ml.

### Lymphocyte subgroup analysis

Complete blood count and serum immunoglobulins were tested, lymphocyte subgroups, T and B cell subgroup analysis were carried out using flow cytometry. Peripheral blood samples were collected into EDTA tubes. Peripheral blood mononuclear cells (PBMC) from patients and healthy control subjects were separated using the density gradient centrifugation method. Then, PBMCs were washed twice. Stained cells were analyzed using a Beckman Coulter Navios (Brea, CA, USA). Standard multiparametric flow cytometry protocols for lymphocyte subset analysis were applied. For lymphocyte subsets, anti-CD3 (FITC), anti-CD4 (APC), anti-CD8 (PC7), anti-CD16+56 (PE), CD19 (A700), anti-TCR alpha-beta (PE), anti-TCR gamma-delta (FITC), CD45RA (FITC), and CD45RO (PE). All antibodies were used at optimal concentrations according to the manufacturers' instructions (Becton Dickinson, San Jose, CA, USA).

### T and B cell subgroup analysis

For T cell subgroup analysis, the panel, including anti-CD4 (FITC), anti-CD8 (PC7), anti-CD31 (PB), anti-CD45RA (APC), and anti-CCR7 (PE) (Beckman Coulter, Brea, CA, USA) was used. For B cell subgroup analysis, the panel including anti-CD19 (PC5.5), anti-CD21 (PB), anti-CD27 (PE), anti-CD38 (APC750), anti-IgD (FITC), and anti-IgM (APC) (Beckman Coulter, Brea, CA, USA), was used*.*

### Lymphocyte activation test

For the lymphocyte activation test, PBMCs were resuspended in complete culture medium consisting of RPMI-1640 supplemented with fetal bovine serum (FBS), 2 × 10^−3^ L-glutamine, and 1% penicillin-streptomycin, stimulated with phytohemagglutinin, and incubated for 48 H. Cells were then stained with anti-CD3 (PC5.5) and anti-CD4 (FITC), anti-CD69 (PE), and anti-CD25 (APC).

### Next-generation primary immunodeficiency (PID) gene panel analysis and Sanger sequencing

Next-generation sequencing was performed using the primary immunodeficiency gene panel (Ion AmpliSeq Primary Immune Deficiency Research Panel v2)^[11]^, which anchors 265 genes. Firstly, genomic DNA was extracted from whole blood with a genomic DNA extraction kit protocol (EZ1 DNA Blood 200 μl Kit, Qiagen, Germany). DNA libraries were prepared with the Nextera DNA Flex kit, and samples were sequenced on the Illumina NextSeq 2000 platform. Variants obtained after gene panel sequencing were analyzed using ION REPORTER version 5.10. Databases open to international access were used to determine the pathogenicity of the variants obtained. We used ExAC (Exome Aggregation Consortium) and gnomAD (The Genome Aggregation Database) databases for comparing allele frequencies of detected nucleotide changes and allele frequencies in healthy individuals; Polyphen, SIFT, and Mutation Taster scores for the determination of pathogenicity of changes in protein caused by the detected nucleotide change. Initial variant interpretation was obtained from VarSome and InterVar. American College of Medical Genetics and Genomics (ACMG) guidelines^[12]^ were followed to interpret pathogenicity of the detected variant, and data from segregation analysis, *in-silico* predictions, structural modelling, evolutionary conservation, and flow cytometric functional data were incorporated.

In order to perform Sanger sequencing analysis, genomic DNA was extracted from 200 μl of blood using the QIamp DNA Blood Kit (Qiagen), according to the manufacturer's instructions. PCR reactions were carried out in a 100 μl reaction volume containing 50 ng of DNA, buffer 10X, 1.5 × 10^−3^ mol/L MgCl2, 0.2 × 10^−3^ dNTPs, 2.5 U AmpliTaq Gold (Applied Biosystems), and 50 pmol of primers designed to amplify all *CD70* coding exons. PCR conditions were as follows: a denaturation step at 95 °C for 5 min; five cycles, 35 cycles at 95 °C for 1 min, 58 °C for 1 min, and 72 °C for 1 min, and a final extension step at 72 °C for 10 min. PCR products were directly sequenced using the appropriate primers and BigDye® Terminator v3.1 Cycle Sequencing Kits (Applied Biosystems). The products were loaded on a 3130 Genetic Analyzer, and the results were analyzed with Sequencing Analysis v.5.2 software.

### *In-silico* analyses: structural modelling and stability prediction

Three-dimensional structural visualization of the CD27/CD70 complex was generated using PyMOL (The PyMOL Molecular Graphics System, v3.1.3. Schrödinger, LLC), from protein structure coordinates from Protein Data Bank (PDB) entry 7kx0. Evolutionary conservation at position 146 was assessed using multi-sequence alignment (UniProt orthologs aligned with Clustal W [v2.1]).

### Functional validation of CD70 mutation by expression analysis

Peripheral blood samples were collected from the CD70 deficient patient and a healthy control subject in EDTA-containing tubes for functional validation. Peripheral blood mononuclear cells (PBMC) were isolated using the density gradient method. Then, PMBC were washed once and incubated with phytohemagglutinin for 2 d in a humidified incubator at 37 °C, with 5% CO_2_. At 48 h of incubation, IL-2 (100 ng/ml) was added. Following stimulation, the cells were collected and washed twice with phosphate buffer saline. Then, cells were stained with phycoerythrin-conjugated anti-CD70 antibody purchased from BD (USA). Stained cells were analyzed for surface CD70 expression using a FACS CANTO II flow cytometer (Becton Dickinson, San Jose, CA, USA).

Statistical analysis was not feasible. This study was conducted in accordance with the Declaration of Helsinki, and the study protocol was approved by Hacettepe University Ethics Committee (Approval Number: GO-21/808; date: 29.06.2021, Approval Number: SBA-2025/1001; date: 09.12.2025). Written informed consent was obtained from the patient's parents.

## Case presentation

An 11-year-old girl was brought unconscious to the emergency department. She was afebrile with marked fatigue and weakness for the last 4 d, and nocturia and vomiting were noted over the last 2 d. Symptoms of infection were absent. She weighed 48 kg (1.1 SDS), and her height was 154 cm (1.3 SDS). She was tachycardic (155 bpm), hypotensive (60/40 mmHg) with a Glasgow Coma Scale of 7. Pupils were anisocoric, papilledema, prolonged capillary refill were present, skin turgor and tonus were decreased. Purpuric lesions of 3 × 4 cm, 2 × 2 cm, and 2 × 1 cm were evident on her shoulder, chin, and palm, respectively (Fig. 1a). History revealed that she had congenital hypothyroidism diagnosed through neonatal screening, and an inexplicable, non-specific erythematous rash intractable to treatment for the past two years. Her parents were first cousins, and her grandparents were using oral antidiabetics for type 2 diabetes.

**Figure 1:**
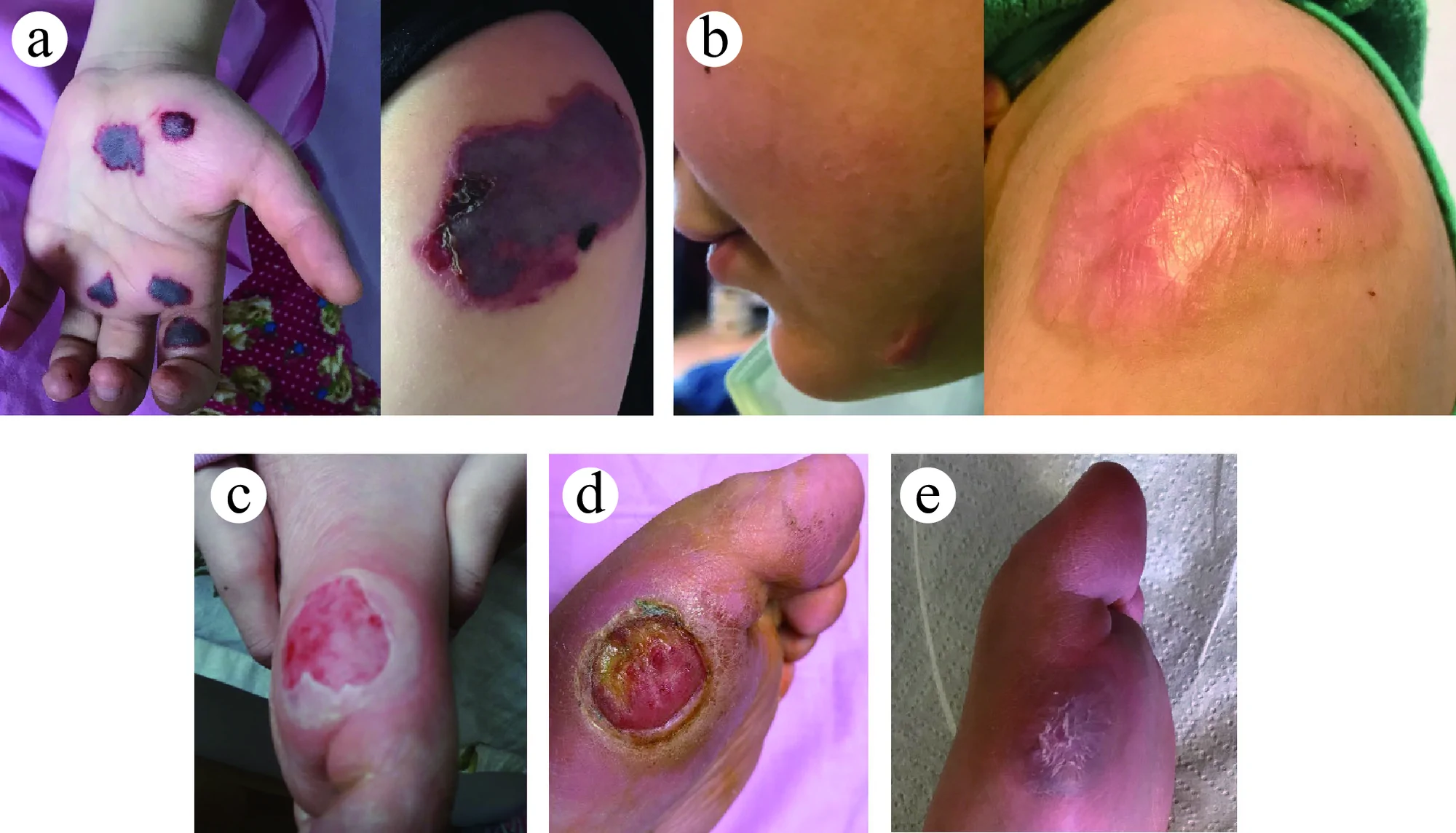
(a) Purpuric lesions on the palm and left shoulder on admission. (b) Purpuric lesions on the chin and shoulder healing with atrophic scar tissue. (c) New bullous lesion on the left metatarsi one month after first admission. (d) Ulcerated foot lesion, three months after first admission. (e) Healed left metatarsi lesion.

Diagnosis of severe diabetic ketoacidosis (DKA) was established, with suspicion of accompanying brain edema (Table 1). She was intubated, adrenaline infusion, and treatment for DKA in accordance with ISPAD Guidelines were initiated^[13]^. Hypertonic saline and mannitol were administered for brain edema. Laboratory evaluations and monitoring indicated multiorgan dysfunction (pancytopenia, respiratory and circulatory failure, complications of severe DKA, i.e., severe rhabdomyolysis and acute renal failure [Table 1]). Despite increasing intravenous insulin for up to 0.7 U/kg/h in the initial 4 h, hyperglycemia and metabolic acidosis did not resolve, and urine output was < 1cc/kg/h; thus, continuous veno-venous hemodiafiltration was started. Adrenal insufficiency was ruled out; thyroid function tests were within normal limits under 37.5 mcg levothyroxine (Table 1). Blood, urine cultures, EBV serology, respiratory viral panel, COVID-19 polymerase chain reaction, and antibodies were negative. Autoantibodies for diabetes were consistent with T1DM. Thyroid autoantibodies and tissue transglutaminase IgA were negative (Table 1).

**Table 1:** Laboratory evaluation on admission and on the last clinical visit (laboratory normative data are provided in parentheses).

	On admission	Last clinical visit
Hemoglobin (gr/dl) (11.5–15.5)	10.9	12.2
Leucocyte (10^3^/μL) (5,000–13,000)	3,400	4,300
Trombocyte (10^3^/μL) (180,000–400,000)	28,000	210,000
ALT (U/L) (< 39)	120	23
AST (U/L) (< 51)	449	40
Blood urea nitrogen (mg/dL) (5–18)	29	9.3
Uric acid (mg/dL) (2.6–6)	8.9	4.3
Creatinine (mg/dL) (0.3–0.8)	2.6	0.5
Sodium (mEq/L) (135–145)	141	139
Potassium (mEq/L) (3.4–4.7)	3.1	4.5
Calcium (mg/dL) (8.8–10.8)	9.4	9.8
Phosphate (mg/dL) (3.2–5.7)	2.8	4.1
Creatine kinase (U/L) (< 145)	51,142	NA
*Coagulation parameters*		
INR (0.8–1.2)	1.5	1.1
aPTT (sn) (22.5–32)	36.2	22.1
Fibrinogen (mg/dL) (180–350)	193	421
d-dimer (mg/L) (0–0.55)	6.6	0.19
*Blood gas*		
pH	6.8	7.38
HCO_3_ (mmol/L)	6.2	23.5
pCO_2_ (mmHg)	27.9	40.5
*Urine sample*		
Density (1,003–1,030)	1,019	
Protein (Negative)	2+	
Blood (Negative)	2+	
Glucose (Negative)	4+	
Ketone (Negative)	3+	
*Endocrine evaluation*		
Blood glucose (mg/dl) (70–100)	> 500	148
Insulin (μIU/ml)	1.5	NA
c-peptide (ng/mL)	1.13	0.1
HbA1c (%) (3.5–5.8)	13.6	6.3
Anti-GAD_65_ (U/mL) (< 1)	1.85	0.1
Anti-IA-2 (IU/L) (< 1)	17.99	0.01
ACTH (pg/mL) (0–46)	692	NA
Cortisol (μg/dL) (6.7–22.6)	39.58	NA
TSH (mIU/mL) (0.4–5.3)	1.5	4.8
FT4 (pmol/L) (7.8–13.7)	12.9	11.3
Anti-TPO (IU/mL) (0–9)	0.3	3.1
Anti-Tg (IU/mL) (0–4)	0.9	0.9
IgA (mg/dL) (67–433)	147	140
TTg IgA (RU/mL) (0–20)	18	Negative

On her second day, she still had papilledema, and pupils were anisocoric, but she could localize pain. Acidosis subsided; inotropes and intravenous insulin (0.1 U/kg/h) could be reduced. Purpuric lesions increased in size, and new lesions appeared. A skin biopsy was performed, revealing significant fibrin thrombi, suggesting vasculopathy (Fig. 2d). Inotropes and continuous veno-venous hemodiafiltration were discontinued, and she could be extubated on the fifth day, when normal urine output was restored. Following extubation, oral feeding and subcutaneous four-dose short-acting human insulin (regular insulin, 1 U/kg/d) were started; dose titration was carried out for optimal glycemic control. After calculation of total daily dose (1.25 U/kg/dose), the insulin regimen was continued as long-acting insulin analog (insulin glargine, once daily, 40% of total daily dose) and rapid-acting prandial insulin (insulin lispro, 3x). Prior to this incident, she was normally functioning without any neurological complaints. However, post-extubation, motor strength was 3/5 in the upper extremities and 2/5 in the lower extremities, accompanied by visible thenar and hypothenar atrophy; maximum motor capability was unsupported sitting. Postural tremor was observed, along with increased deep tendon reflexes and sensory loss in the lower extremities. Electromyography revealed polyneuropathy in both motor and sensory nerves. These findings were associated with critical illness myo-neuropathy, prompting the initiation of physiotherapy. Following a two-week period, the patient exhibited improvements in pancytopenia, elevated transaminases, and renal function tests, leading to her discharge with a basal-bolus insulin regimen.

**Figure 2:**
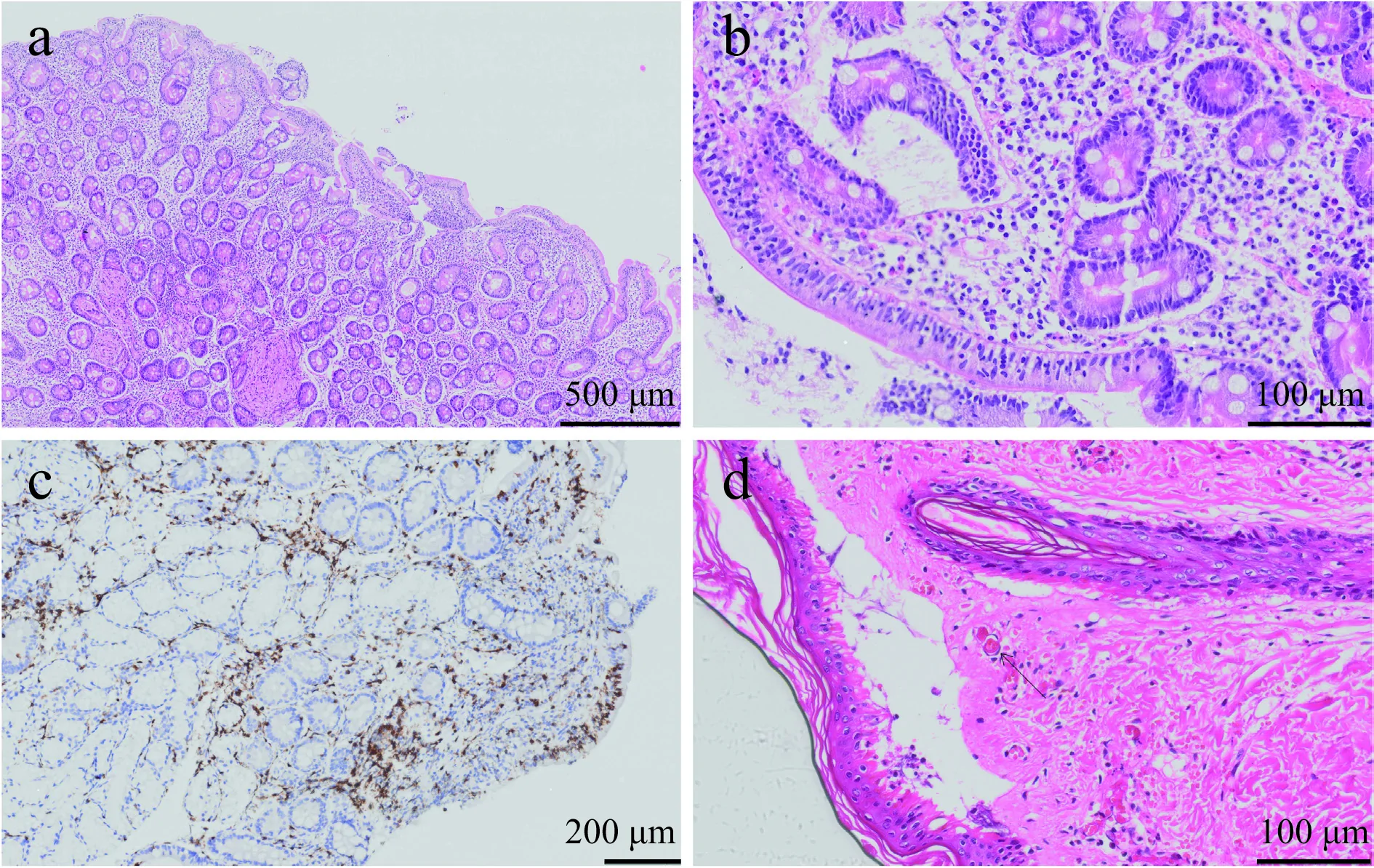
(a) Villous atrophy and crypt hyperplasia in endoscopic bulbus biopsy (Hematoxylin and Eosin, scale bar: 500 µm). (b) Increased intraepithelial lymphocytes in endoscopic bulbus biopsy (Hematoxylin and Eosin, scale bar: 100 µm). (c) Increased intraepithelial lymphocytes evaluated with CD3 immunohistochemistry (scale bar: 200 µm). (d) Skin biopsy with fibrin thrombi in dermal capillary vessels (Hematoxylin and Eosin, scale bar: 100 µm).

One month later, the initial purpuric lesions resolved with atrophic scar tissue (Fig. 1b), and a new bullous lesion appeared on the medial left metatarsus (Fig. 1c). The patient complained of bilateral burning and jabbing pain in the lower extremities. Gabapentin was initiated for neuropathic pain. Three months later, the ulcerated foot lesion on the left metatarsal worsened despite an oral antibiotic regimen, requiring initiation of intravenous antibiotics (Fig. 1d). Cellulitis and osteomyelitis were absent, and the swab culture did not yield any pathogens. The patient was discharged with minimal healing of the lesion. She participated in diabetes education, learned and regularly practiced carbohydrate counting, and correctly applied correction doses, and we followed her closely, communicating with the family nearly every day to help regulate the unexpected fluctuations in blood glucose. However, she experienced a dramatic increase in blood glucose excursions. Following her initial admission, despite being compliant with the treatment and close follow-up, she had multiple episodes of diabetic ketosis and ketoacidosis (Fig. 3). T1DM was poorly controlled, and all these episodes developed rapidly within hours without identifiable precipitating causes. In the subsequent year, tissue transglutaminase IgA was tested positive during an asymptomatic phase, for which an endoscopic biopsy was performed. Pathology indicated celiac disease (Marsh type-3b, Fig. 2a–c), and she was advised to follow gluten-free diet. Hospitalization for acute severe DKA, accompanied by multiorgan dysfunction, and subsequent follow-up characterized by recurring rapid-onset DKA episodes, unexplained skin lesions, and celiac disease prompted an evaluation of an underlying condition. Immunophenotype analysis and next-generation gene sequencing analysis revealed a previously reported^[8]^ homozygous missense variant in *CD70* (c.437G > T [p.S146I]) (Fig. 4a).

**Figure 3:**
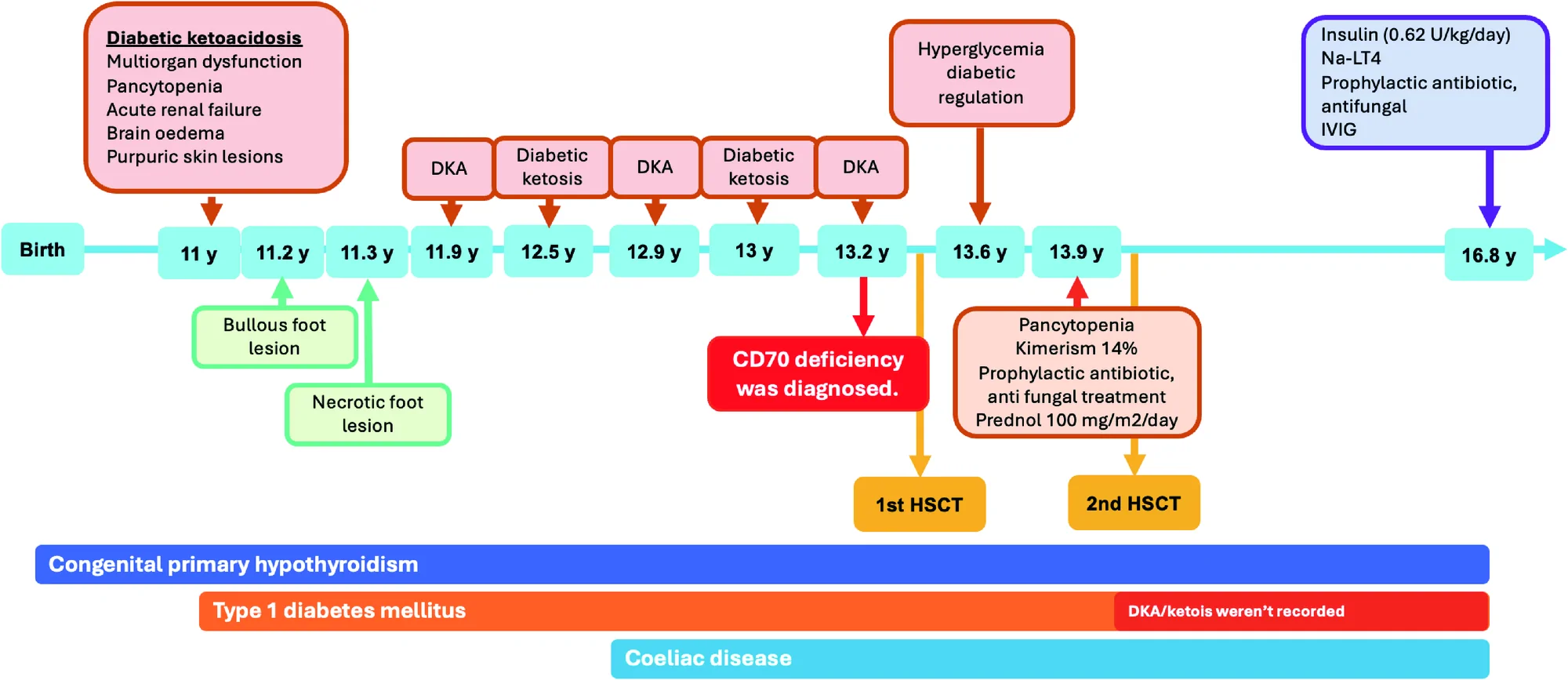
Timeline for the course of symptoms in CD70 deficiency. DKA: diabetic ketoacidosis, HSCT: hematopoietic stem cell transplantation, IVIG: intravenous immunoglobulin, y: years, Na-LT4: Na-levothyroxine.

**Figure 4:**
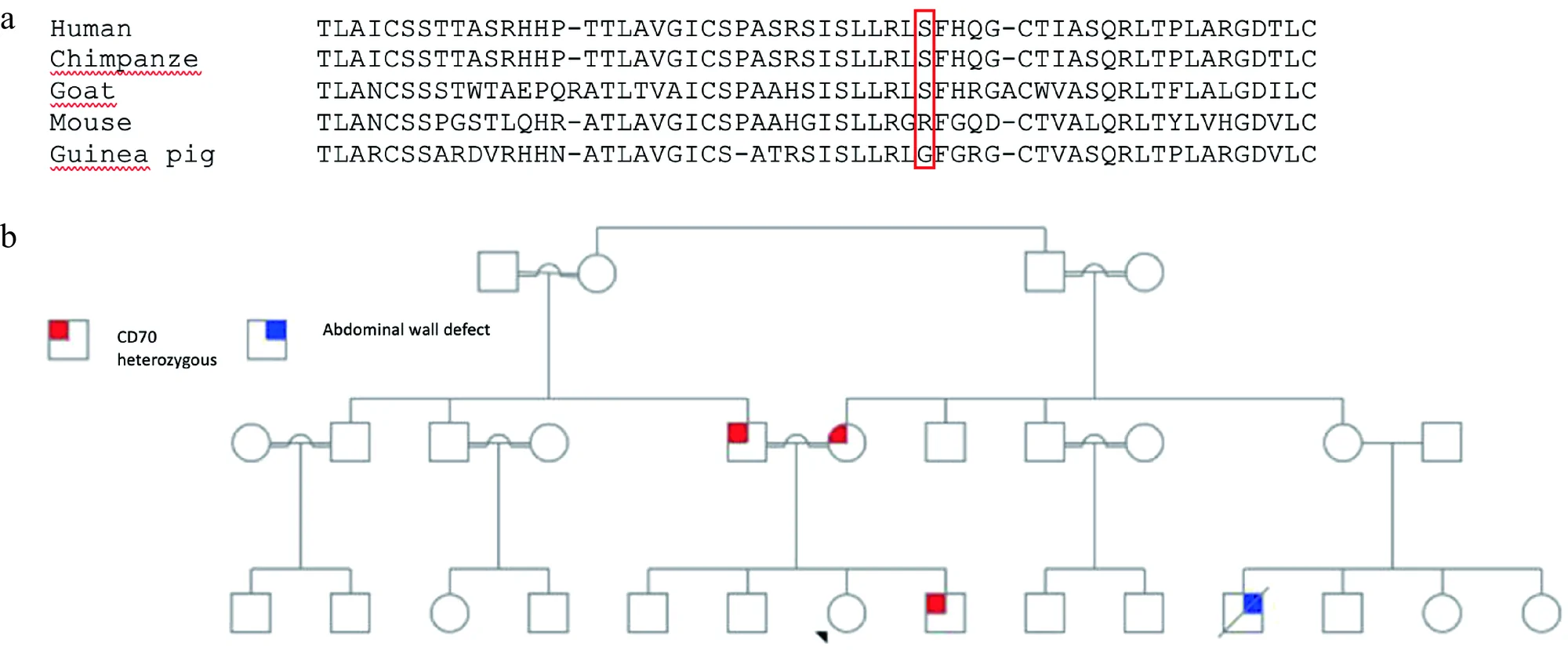
(a) Multiple sequence alignment of CD70 orthologs from five species (human, chimpanzee, goat, mouse, and guinea pig) generated using Clustal W (v2.1). Position p.S146 in the human sequence is indicated by a red rectangle. (b) Pedigree of the proband*.* Parents and the 11-year-old brother are heterozygous carriers of the mutation.

## Results

### Immunophenotype findings

Initial laboratory evaluation showed that complete blood count, serum immunoglobulin levels, and lymphocyte subsets were within age-adjusted reference ranges. Detailed immunophenotype analysis demonstrated mildly decreased marginal zone B cells, transitional B cells, and plasmablasts. Among T-cell subsets, CD4+ TEMRA cells were increased, and CD8+ TEMRA cells were also elevated. The proportion of TCR α*β*+ CD4−CD8− T cells was 8%. Immunophenotype evaluation revealed the possibility of combined T and B cell deficiency or an underlying immune dysregulation. Based on these findings, genetic evaluation was performed (Table 2). Monthly intravenous immunoglobulin replacement therapy (400 mg/kg) was initiated, and HSCT was subsequently performed. Follow-up after HSCT revealed that marginal zone B cells, transitional B cells, and plasmablasts were increased when compared to pre-transplant values. Effector memory CD4+ T cells were decreased, and CD8+ TEMRA cells remained increased during follow-up. The proportion of TCR α*β*+ CD4−CD8− T cells decreased after transplantation.

**Table 2:** Laboratory examination obtained from a peripheral blood sample on admission, one year, and 33 months after HSCT.

		On admission (11 year 3 months)	One year after HSCT	33 months after HSCT
	Leukocytes (cells/mm^3^)	4,700 (4,500–13,500)	5,290 (4,500–13,500)	5,820 (4,500–13,500)
	Absolute lymphocyte count (cells/mm^3^)	2,600 (1,500–5,000)	2,650 (1,500–5,000)	3,140 (1,500–5,000)
	Absolute neutophil count (cells/mm^3^)	1,700 (1,800–8,000)	2,210 (1,800–8,000)	2,220 (1,800–8,000)
Serum immunoglobulin^[14]^	IgG (mg/dL)	988 (835–2,094)	1,090*	1,260*
	IgA (mg/dL)	147 (67–433)	28.3 (100−447)	29.7 (100−447)
	IgM (mg/dL)	168 (47−484)	219 (75−448)	119 (75−448)
Antibody response	Isohemaglutinins Anti-A	1/256	NA	NA
	Anti-B	Negative	NA	NA
	Anti HBs (mIU/mL)	20.44	NA	367.3
Lymphocyte subsets^[15]^	CD3 (%)	68(60−76)	79 (56−84)	70 (56−84)
	CD4 (%)	32(31−47)	25 (31−52)	28 (31−52)
	CD8 (%)	31(18−35)	49 (18−35)	38 (18−35)
	CD16+56 (%)	7(4−17)	2 (3−22)	2 (3−22)
	CD19 (%)	23(13−27)	18 (6−23)	28 (6−23)
	CD45RA (%)	57(46−77)	NA	NA
	CD45RO (%)	31(20−46)	NA	NA
	TCR Alpha-Beta (%)	67	NA	NA
	TCR Gamma-Delta (%)	4	NA	NA
	TCR Alpha-Beta + CD4-CD8−	8	4	2
B cell subsets^[16]^	CD20^+^ (%)	13	NA	21
	Memory (CD19^+^CD27^+^) (%)	12.7 (13.3−47.9)	NA	12.3 (13.3−47.9)
	Switched-memory(CD19^+^CD27^+^IgD^−^) (%)	10.5 (4.6−18.2)	NA	7.4 (4.6−18.2)
	Marginal zone (CD19^+^CD27^+^IGD^+^) (%)	2.2 (4.6−18.2)	NA	4.9 (4.6−18.2)
	Naive (CD19^+^CD27^−^IGD^+^) (%)	82.9 (51.3−82.5)	NA	84.4 (51.3−82.5)
	Activated (CD19^+^CD38^−^CD21^low^) (%)	1.1 (2.7−8.7)	NA	0.3 (2.7−8.7)
	Plasmablast (CD19^+^CD38^+high^ IgM^−^) (%)	0.3 (0.6−6.5)	NA	1 (0.6−6.5)
	Transitional (CD19^+^CD38^+high^ IgM^high^) (%)	1.1(1.4−13)	NA	2.8 (1.4−13)
T cell subsets^[17]^	CD4^+^ cell rate (%)	35 (29−59)	NA	27 (29−59)
	Naive T_H_ (CD4^+^CCR7^+^CD45RA^+^) (%)	77.4 (57.1−84.9)	NA	55.5 (57.1−84.9)
	Central memory T_H_ (CD4^+^CCR7^+^CD45RA^−^) (%)	4.7 (11.3−26.7)	NA	0.4 (11.3−26.7)
	Effector memory T_H_ (CD4^+^CCR7^−^CD45RA^−^) (%)	5.5 (3.3−15.2)	NA	2.6 (3.3−15.2)
	TEMRA (CD4^+^CCR7^−^CD45RA^+^) (%)	12.2 (0.4−2.6)	NA	41.4 (0.4−2.6)
	RTE (CD4^+^CD31^+^CD45RA^+^) (%)	57 (31−81))	NA	37 (31−81)
	CD8^+^ cell rate (%)	35 (19−29)	NA	46 (19−29)
	Naive T_C_ (CD8^+^CCR7^+^CD45RA^+^) (%)	34.5 (28.4−80.6)	NA	19.8 (28.4−80.6)
	Central memory T_C_ (CD8^+^CCR7^+^CD45RA^−^) (%)	0.3 (1.0−4.5)	NA	0.1 (1.0−4.5)
	Effector memory T_C_ (CD8^+^CCR7^−^ CD45RA^−^) (%)	9.8 (6.2−29.3)	NA	0.4 (6.2−29.3)
	TEMRA (CD8^+^CCR7^−^CD45RA^+^) (%)	55.2 (9.1−49.1)	NA	79.6 (9.1−49.1)
Complements	C3 Complement (mg/dl)	90.2	174	NA
	C4 Complement (mg/dl)	19.3	36	NA
	*After IVIG replacement			

### PID-next generation-sequencing panel analysis and Sanger sequencing

Next-generation gene sequencing analysis for primary immunodeficiency revealed a previously reported^[8]^ homozygous missense variant in *CD70* (c.437G > T [p.S146I])*.* This variant was confirmed by Sanger analysis, and segregation analysis revealed that parents and the 11-month-old brother were heterozygous for the same mutation (Fig. 4b).

### *In-silico* analyses: structural modelling and stability prediction

To further clarify the pathogenic significance of this missense variant, a detailed *in-silico*, structural, evolutionary, and receptor-binding analysis was performed. Multi-species alignment showed that the Serine residue at position 146 is conserved in chimpanzee and goat (Fig. 4a). Mouse and guinea pig have a shorter CD70 protein. The residue corresponded to an Arginine (R) in mouse and a Glycine (G) in guinea pig (Fig. 4a). From a steric perspective, little difference between Serine (human) and Glycine (guinea pig) was expected, as both are amino acids with small sidechains; however, Arginine is considerably larger and is positively charged.

The wild-type structure (left) has a polar serine (Ser) residue at position 146, whereas the mutant structure (right) has a hydrophobic isoleucine (Ile) at the same location. The crystal structure of the CD27/CD70 complex has recently been published^[18]^. From a protein structure perspective, this structure reveals CD70 as a homocomplex of three TNF-like domains forming a trimeric core (described as a 'blooming flower'). This CD70 trimer is complexed with three CD27 molecules, each interfacing a groove provided by pairs of CD70 molecules within the trimer, such that a 3:3 CD27/CD70 complex is formed (Fig. 5a); the CD70 trimer might be projected from the surface of an antigen-presenting cell, and the CD27 molecules projected from the surface of a T cell. S146 is located within each CD70 molecule at the interface with CD27 (Fig. 5b). *In-vitro* binding experiments have shown that the S146D variant of CD70 is no longer able to interface CD27, likely the result of a proposed steric clash accompanying substitution of a serine residue with a larger aspartic acid^[18]^. Substitution of a similarly large isoleucine residue at this position might also prevent binding due to steric clash issues. Alternatively, the hydrophobic nature of the S146I substitution might result in CD70 protein folding issues. PyMOL was used to visualize both structures (The PyMOL Molecular Graphics System, v3.1.3. Schrödinger, LLC) (Fig. 5b).

**Figure 5:**
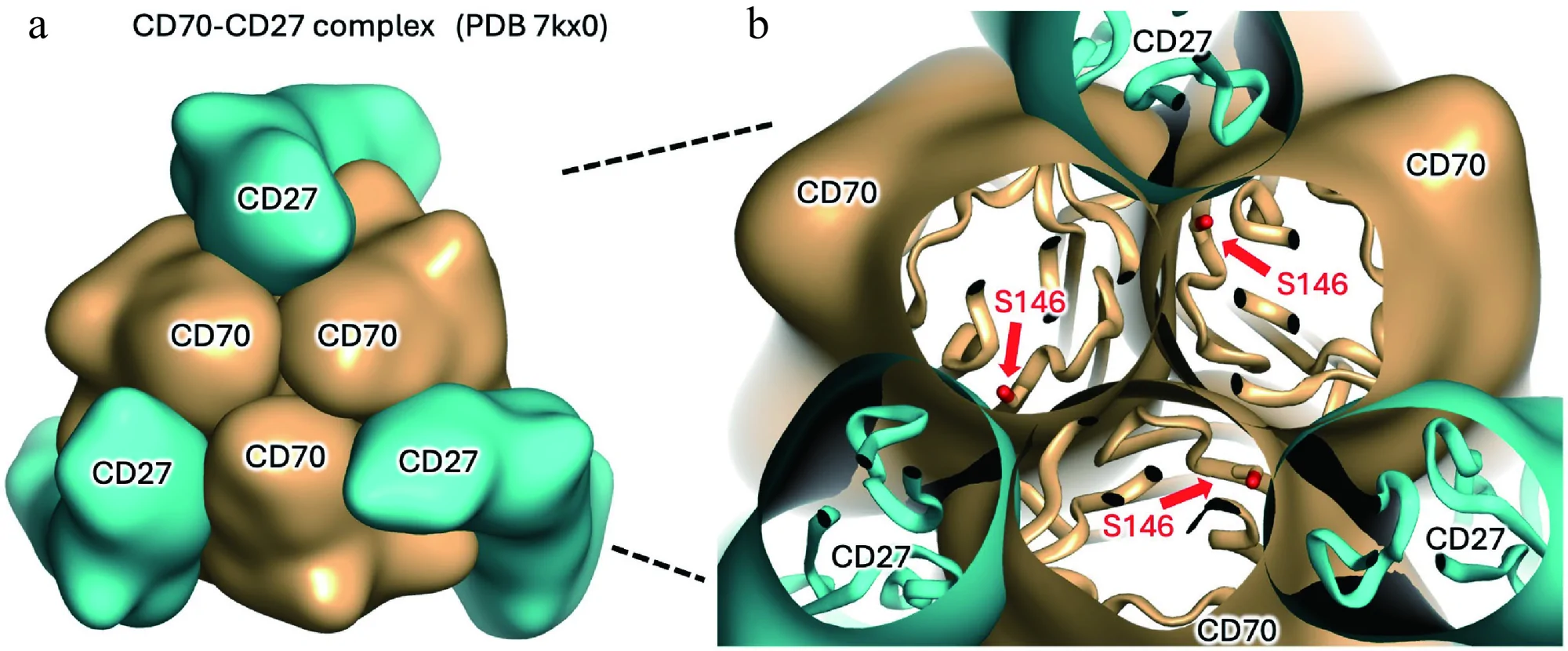
Structural depiction of the CD70-CD27 complex. (a) Smoothed surface representation showing a trimer of CD70 molecules (tan surfaces, as might be projected from the exterior of an antigen-presenting cell), bound along protomer interfaces by three molecules of CD27 (cyan surfaces, as might be projected from the surface of an adjacent T-cell). (b) Cut-away section revealing polypeptide folds (ribbons) and residue S146 of CD70 shown as sticks. S146 is located at the surface of each CD70 protomer, adjacent to the CD27 binding site. The image was generated using Pymol using coordinates from Protein Data Bank entry 7kx0^[18]^.

### Functional validation of CD70 mutation by expression analysis

CD70 expressions tested both baseline (unstimulated) (MFI of HC: 117 vs P: 39.8) and following stimulation (MFI of HC: 519 vs P: 281), were lower in the patient compared to the healthy control (Fig. 6).

**Figure 6:**
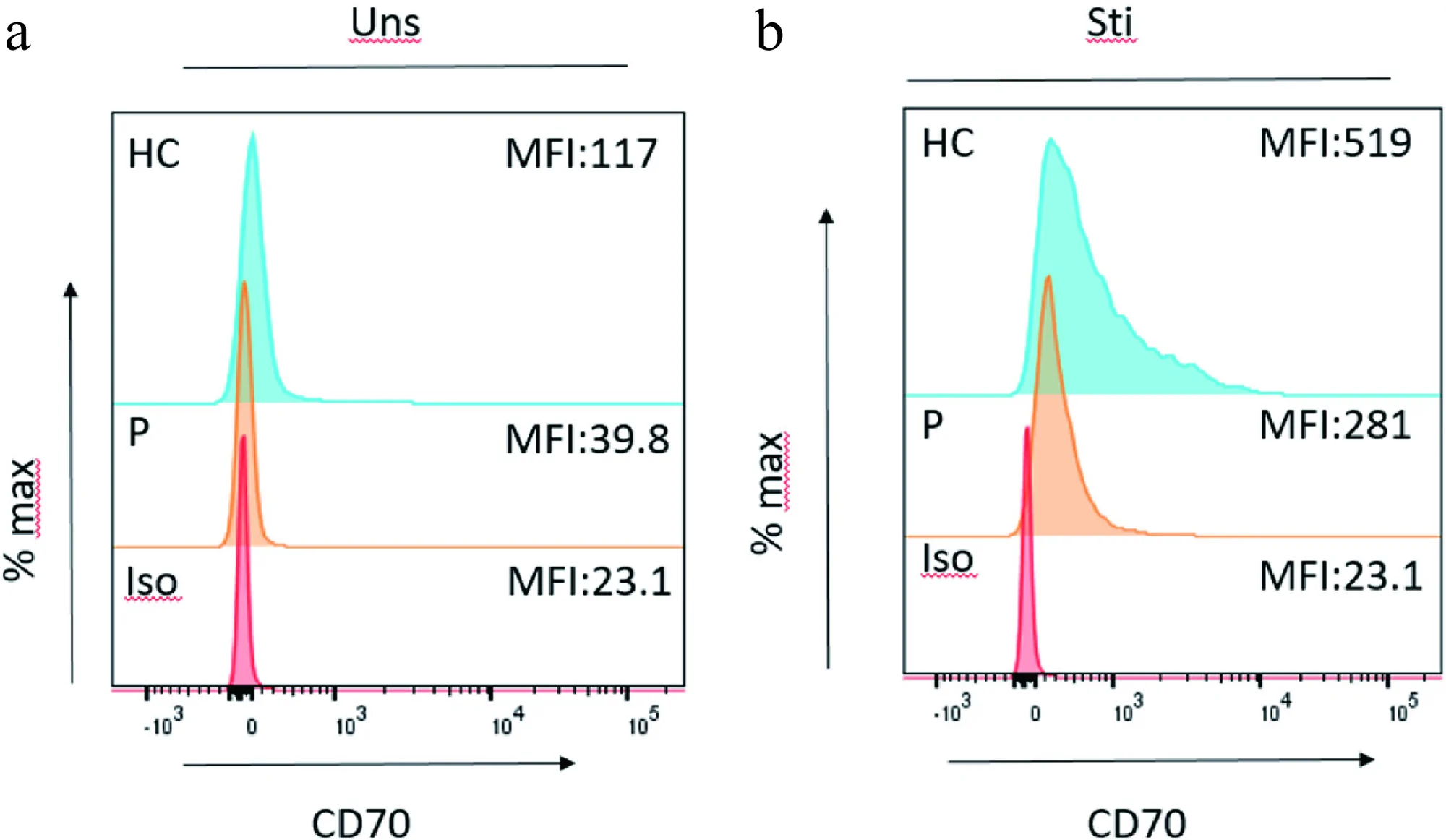
Flow cytometric CD70 expression in unstimulated (uns) and stimulated (sti) cells. Histograms show CD70 expression in healthy control (HC, blue), patient (P, orange), and isotype control (Iso, red). (a) Unstimulated condition: CD70 expression was minimal in the patient and control, with lower median fluorescence intensity (MFI) in the patient (MFI = 39.8) compared to the healthy control (MFI = 117). (b) Following stimulation with phytohemaglutinin (PHA) and interleukin (IL)-2, CD70 expression was markedly induced, with higher MFI values observed in healthy control (MFI = 519) relative to patient (MFI = 281). These findings indicate impaired upregulation of CD70 in the patient cells upon activation compared to control.

The detected missense variant in *CD70* (c.437G > T [p.S146I]) is very rare in population databases. Stability prediction studies revealed possible steric clash or disruption in protein folding, while functional studies demonstrate reduced CD70 surface expression, with no reported homozygotes. Considering segregation analysis, compatible clinical and immune phenotype, as well as the previously reported case with the same mutation^[8]^, CD70 deficiency was diagnosed.

### Hematopoietic stem cell transplantation characteristics

Two and a half years after T1DM diagnosis, she underwent HSCT from a 10/10 HLA-matched sibling, utilizing a myeloablative conditioning regimen of busulphan (12.8 mg/kg) and fludarabine (160 mg/m^2^). Cyclosporin A (CsA; 2 x 1.5 mg/kg/d, dosage adjustments were made to achieve a target level of 150–200 µg/L) and methotrexate (10 mg/m^2^ on day +1, +3, and +6) were used as prophylaxis for graft vs host disease. Starting from three months post-HSCT, CsA was tapered off and discontinued at four and a half months post-HSCT. Successful neutrophil engraftment was achieved on +16 d, and thrombocyte engraftment was achieved on +20 d (chimerism analysis was 74% donor profile +1 month). During follow-up, donor chimerism decreased, and seven months post-HSCT, secondary graft failure developed (chimerism analysis was 3% donor profile). Eight months after the first HSCT, a second HSCT was performed with a reduced toxicity regimen including treosulfan (42 mg/m^2^), fludarabin (150 mg/m^2^), and anti-thymocyte globulin (30 mg/kg). CsA (2 x 1.5 mg/kg/day; dosage adjustments were made to achieve a target level of 150–200 µg/L) and mycophenolate mofetil (3 x 15 mg/kg/day) were used as prophylaxis for graft vs host disease. Mycophenolate mofetil was discontinued three months post-HSCT, while CsA was tapered off starting at three months and was discontinued at three months post-HSCT. Following the second HSCT, stable full donor chimerism (≥95% donor profile) was achieved. Following engraftment, glycemic control improved (median HbA1c: 8.1% [7.6–8.6] vs 6.5% [5.8–7.0]), and the total daily insulin dose decreased from 0.85 to 0.62 U/kg/day (Fig. 7). She is monitored quarterly in the diabetes clinic as part of standard follow-up, with no need for additional clinical visits, and has not experienced any new episodes of DKA or ketosis 33-months after second HSCT (Fig. 3). On the last clinical visit, fasting c-peptide remained low, while anti-GAD_65_ and anti-IA-2 were decreased to undetectable levels. She has a normal growth trajectory (Fig. 8) while adhering to a gluten-free diet. Her latest tissue transglutaminase IgA levels are negative, and the reintroduction of gluten is being contemplated.

**Figure 7:**
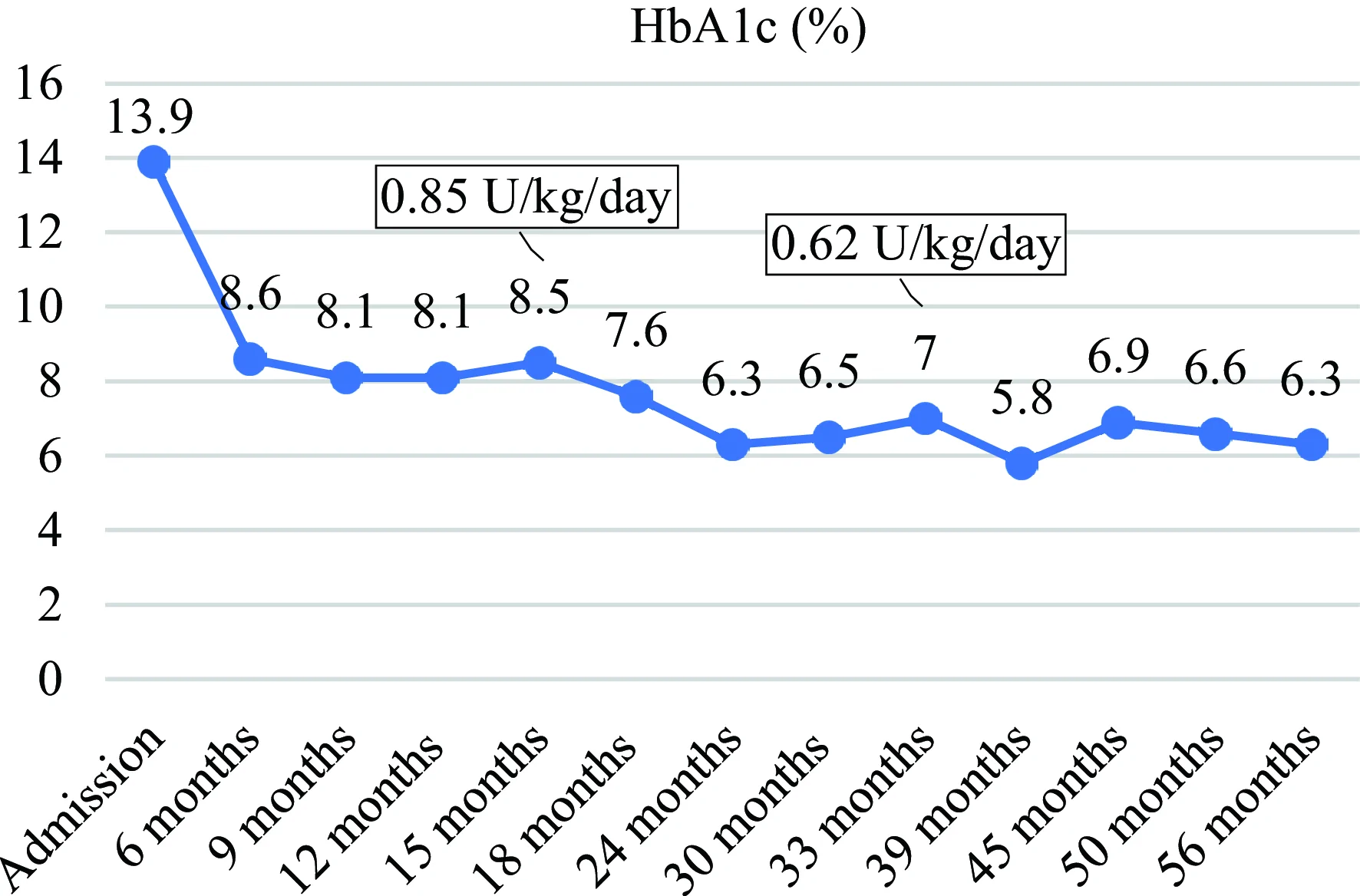
Change in HbA1c. Months denote time spent following the first admission. The first HSCT was performed at 18 months, and the second HSCT was performed at 24 months.

**Figure 8:**
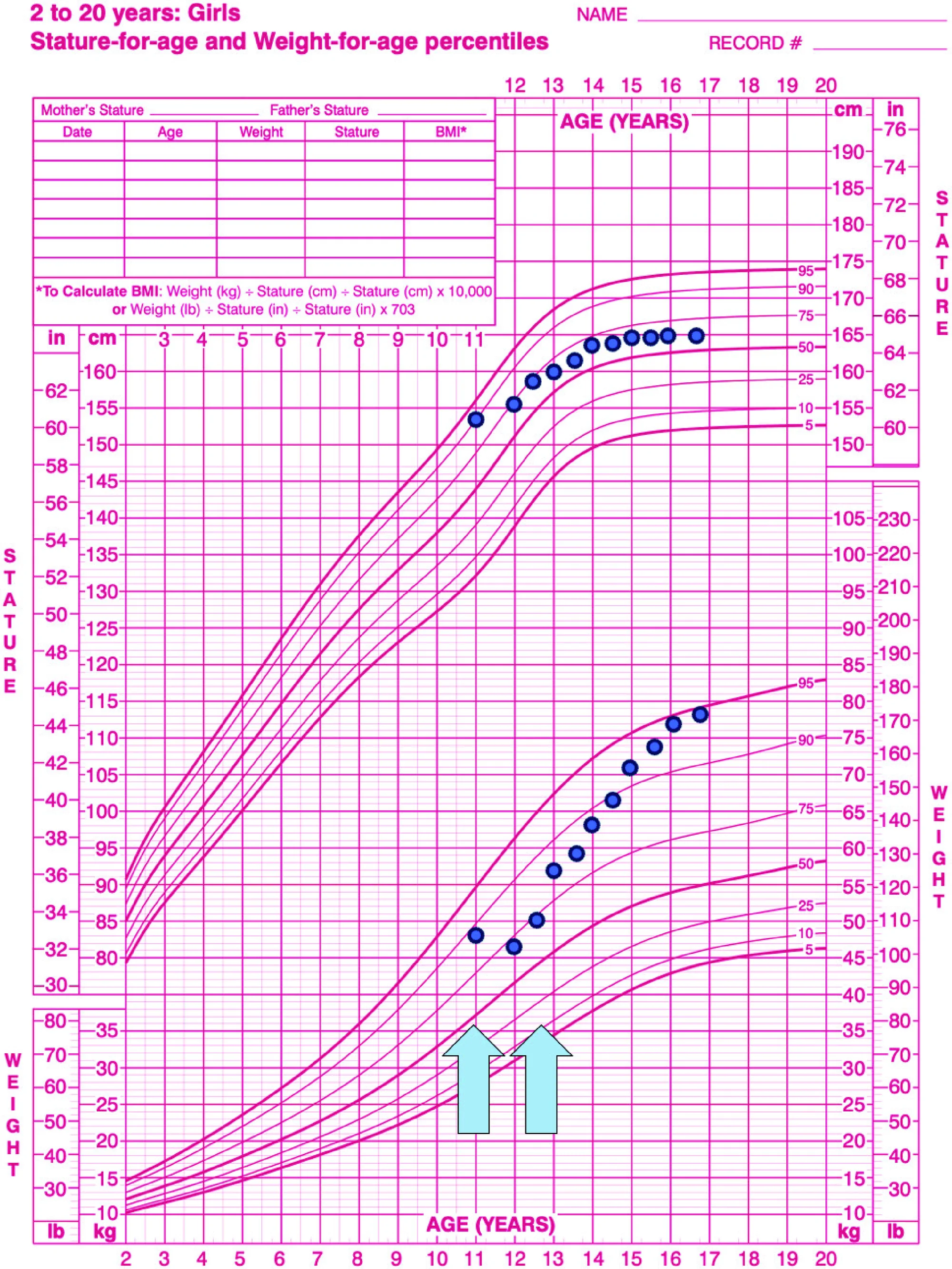
Shows the growth chart of the case with CD70 deficiency. The first arrow depicts her presentation with severe diabetic ketoacidosis, and the second arrow depicts the diagnosis of coeliac disease.

## Discussion

This case represents the first documentation of T1DM associated with a homozygous missense mutation in the *CD70* in humans. The onset of T1DM was severe with rapidly deteriorating DKA and multiorgan failure. This emphasizes the necessity for evaluating the underlying pathophysiology in patients with T1DM displaying unusual clinical manifestations. Moreover, it underscores the crucial role of intact CD27/CD70 co-stimulation in preventing pancreatic * β *-cell autoimmunity, establishing a foundation for preclinical research to clarify the pathophysiology of T1DM, thereby facilitating targeted immunotherapy trials for its prevention and treatment.

Patients with specific IEIs, especially those with immune dysregulation, may offer insights into the pathophysiology of autoimmune disorders like T1DM. Historically, the identification of *FOXP3* mutations in IPEX syndrome, where T1DM represents a frequent clinical feature, was pivotal in revealing the essential role of CD4^+^FOXP3^+^ Tregs in maintaining immune self-tolerance and homeostasis in humans, ultimately paving the way to new immunotherapy trials in T1DM^[2,19]^. It was later established that stable expression of *FOXP3,* which is largely determined by epigenetic demethylation of the Treg-specific demethylation region, regulates Treg function^[19]^. Whether Tregs are a phenotypically and functionally stable population is under debate^[19]^. However, Treg stability is highly dependent on co-stimulatory signals, which are important for Treg activation, survival, expansion, and acquisition of effector functions upon antigen recognition.

Interactions of the ligand–counterligand pair, CD27 and CD70, provide signals for T and B lymphocyte activation, critically regulating both adaptive and innate immune responses. Signaling through CD27 enhances lymphocyte proliferation, survival, differentiation into long-lived memory cells, and cytotoxic effector function, while also modulating activation-induced apoptosis, particularly in CD8^+^ T cells and natural killer (NK) cells. Through these mechanisms, the CD27/CD70 co-stimulatory pathway contributes to effective antiviral, antitumor, and antibacterial immunity and plays an essential role in maintaining immune homeostasis and limiting autoimmunity^[20]^.

The CD27/CD70 interactions are primarily regulated by expression of CD70, which is induced by Toll-like receptor (TLR), CD40, and/or antigen receptor signaling. CD70 is a surface protein bound to activated antigen-presenting cells (APCs), including dendritic cells, B lymphocytes, and activated T lymphocytes^[21,22]^. CD70 interacts with CD27 on T lymphocytes and provides co-stimulatory signals in addition to T cell receptor (TCR) engagement. CD27 signals through NF-kappaB (NFκB) and c-Jun N-terminal kinase (JNK) pathways via TRAF2 and TRAF5. CD27 signaling stimulates naive CD4 T lymphocytes to differentiate into IFNγ producing T helper 1 (Th1) cells by enhancing the Th1-specific transcription factor T-bet. Soluble CD27 can be cleaved from the membrane-bound form on activated T lymphocytes through the action of matrix metalloproteinase and binds to CD70 on B and T lymphocytes. CD70 also has signaling properties after interacting with CD27, and the phosphoinositide 3-kinases and MEK pathways are involved in its signaling^[20]^. It has also been demonstrated that CD27/CD70 co-stimulation in the thymus rescued developing FOXP3^+^CD4 Treg cells from apoptosis and thereby promoted the generation of Treg cells by enhancing positive selection of these cells in the thymus^[23,24]^.

Interestingly, both increased and decreased CD27/CD70 interactions were associated with various autoimmune pathologies^[9]^. Blockade of the CD27/CD70 pathway was shown to ameliorate disease manifestations in animal models, including murine collagen-induced arthritis and experimental colitis. CD70^+^CD4^+^ T lymphocytes were found to be overexpressed in individuals diagnosed with systemic lupus erythematosus^[20]^. CD70 on naive and memory CD4^+^ T lymphocytes was upregulated in patients with rheumatoid arthritis when compared to healthy controls^[20]^. What's more, in murine models, delivering anti-CD70 antibodies resulted in the amelioration of rheumatoid arthritis and a reduction in autoantibody levels^[25]^. Studies have explored the CD27/CD70 pathway in animal models of autoimmune encephalomyelitis and demonstrated that anti-CD70 antibodies exhibit preventative effects^[26]^. On the other hand, several studies have shown that the CD27/CD70 pathway may have a suppressive effect on immune responses. In a mouse model of experimental autoimmune encephalomyelitis, constitutive CD27 signals via the JNK pathway were shown to impede the activity of Th17^[20]^. *In vivo* studies on murine models point out that CD27/CD70 co-stimulation inhibits the generation of competent Th17 effector cells, ameliorating Th17 cell-dependent inflammation and autoimmunity^[23]^. It was proposed that CD70 has an active role in regulating suppressive Treg activity by providing direct co-stimulatory signals to responder T cells. An imbalance between effector and regulatory TH subsets, particularly Th17 effector cells, is central to islet inflammation and progression to T1DM. In this context, dysregulation of CD27/CD70 signaling may contribute to Th17-driven inflammation, facilitating loss of immune tolerance observed in T1DM.

CD70 deficiency, characterized by increased susceptibility to bacterial and viral infections, impaired humoral immunity, and hypogammaglobulinemia, results in combined immunodeficiency. Autoinflammatory symptoms of recurrent fever, aphthous stomatitis, pharyngitis, and cervical adenitis prior to presenting symptoms were identified in 40% of patients with CD70 deficiency^[8]^. Behçet's disease and alopecia areata were also reported^[27]^. The accumulation of autoinflammatory symptoms suggested a regulatory effect of CD27/CD70 signaling on fine-tuning of immune responses^[8]^. T1DM was not previously reported in human subjects with CD70 deficiency, but the effect of the CD27/CD70 axis on T1DM was investigated by Ye et al. on NOD mice^[9]^. CD70 ablation in NOD mice resulted in increased insulitis scores as well as aggressive onset of T1DM, and it was concluded that intact CD27/CD70 co-stimulation favors tolerogenic over pathogenic response in T1DM^[9]^. Alkhamis et al. studied the impact of a combination of CD25, CD70, and CD8 antibodies on islet inflammation and glycemic control in NOD mice^[28]^. They have shown that this antibody combination reduced insulitis, improved islet structure, reversed hyperglycemia, and conferred protection against T1DM for up to 200 d in NOD mice with new-onset T1DM. In NOD mice with advanced T1DM, this antibody combination was shown to improve the blood glucose profile and reduce islet infiltration, preserving morphology^[28]^. This discrepancy between the consequences of CD70 blocking via targeted genes vs antibodies was previously discussed by Coquet et al. It was suggested that this difference in results may be due to the genetic background of the murine models, use of CD70 blocking antibodies rather than gene-targeted mice, or a combination^[23]^. Although data is quite limited, current literature, and our experience demonstrate the significance of intact CD27/CD70 co-stimulation in the preservation of pancreatic * β *-cell function.

The pathogenicity of the detected missense *CD70* variant was confirmed by Sanger analysis, segregation analysis, *in-silico* analyses, structural modelling, and stability prediction tools, in that the S146I variant of CD70 was no longer able to interface CD27. The reduction in CD70 expression in our patient, attributable to the missense variant, was demonstrated in comparison to controls, even though it was not a complete loss of expression. When we analyzed the amino acid conservation in the mutated position (146) in species, it is conserved in human, chimpanzee, and goat, but not in guinea pig and mouse. Unfortunately, the functional relevance of this substitution in mouse remains uncertain in the absence of structural data for the mouse CD27/CD70 complex, particularly given the broad sequence differences amongst species (human: 193 amino acids, mouse: 195 amino acid and guinea pig: 221 amino acids in length). We prefer not to be overly speculative as to the significance of this finding.

The current case manifested later (at 11 years) than previously reported CD70-deficient patients in the literature (3.4 years [six months to nine years]). Despite the most common clinical features of CD70 deficiency being EBV-related, both EBV serology and copy number were negative in the current case. Autoinflammatory symptoms were detected in 5/16 of the previously reported cases^[8]^. To revise the follow-up of the patient with the same variant^[8]^, we contacted the team following that patient. We were informed that the patient developed EBV-associated disease during the first decade of life, was diagnosed with celiac disease and interstitial lung disease later in the fifth decade, and continues to be followed without HSCT, into her fifth decade, with no evidence of endocrine involvement. These show that both immune deficiency and autoimmune disease are manifest in the previously reported patient, suggesting a regulatory T cell defect caused by the same mutation.

As seen, the clinical course of our patient and the previously reported patient^[8]^ with the same mutation are distinctive. No clear genotype-phenotype correlation has been established in CD70 deficiency in that age of onset, severity, and characteristics of symptoms are different in CD70-deficient patients with the identical mutation from the same family^[8]^. So far, no specific disease biomarkers exist, so clinical awareness is crucial in patients with signs of autoinflammation, especially with atypical presentation or unusual/lack of response, where CD27/CD70 defects should be considered.

The timing and treatment strategies for CD70 deficiency are highly variable due to significant phenotypic diversity. HSCT is essential for treating individuals with severe immunodeficiency, with an increased risk of malignancy. In individuals with a milder phenotype, as in our case, the decision and timing of HSCT are determined by the disease's impact in relation to the procedure's adverse effects. In the study by Ghosh et al., patients with CD27 and CD70 deficiency were evaluated, and 8/16 of CD70-deficient patients underwent HSCT at a median age of ten years. At a median follow-up of two years, mortality was 12%, mostly attributed to complications of infection. Researchers did not express their views on the timing of HSCT in CD70-deficient patients; however, they strongly advocated for the consideration of curative HSCT in CD27-deficient patients with relatively mild disease manifestations, as the initial malignant event results in high mortality, and event-free survival post-HSCT is excellent^[8]^. In the current patient, HSCT was performed from a 10/10 HLA-matched sibling, and she has been monitored for 33 months without any complications. As mentioned, during the initial HSCT, engraftment was achieved, however donor chimerism gradually decreased, and in the end, donor and graft failure (donor chimerism <5%) developed. The second HSCT was successful and lasting. Graft failure in IEIs is not uncommon, and its frequency depends on risk factors (mainly donor type, HLA compatibility, and source of stem cells). It may be seen as frequent as 10%, which may increase up to 47%, according to features of HSCT^[29−33]^.

Evidence indicates that hematopoietic stem cells can induce central and peripheral immune tolerance^[34]^, rendering HSCT a potentially valuable tool in the treatment of immune-mediated diseases^[35]^. HSCT was indicated for IEI in our case. However, it raised a new question about whether HSCT can have a positive impact on glycemic control or gluten sensitivity, even 2.5 years postdiagnosis. Insulin independence could not be achieved in the current case, however glycemic control improved significantly. Prior to HSCT, despite proper diabetes education, carbohydrate counting, and close monitoring, she experienced frequent episodes of blood glucose excursions that rapidly progressed into diabetic ketosis and ketoacidosis without discernible precipitating conditions. However, following the achievement of stable donor chimerism with the second HSCT, she has been followed up for 33 months without any episode of DKA or ketosis. In addition, anti-GAD_65_ and anti-IA decreased to undetectable levels, indicating immune reconstitution; however, fasting c-peptide remained low. Studies on patients with established T1DM indicated a reduced total daily insulin requirement, and in some cases, insulin independence, along with a significant rise in the mean area under the curve for C-peptide, when HSCT is performed promptly after the diagnosis of T1DM^[36−38]^. Despite a relapse rate of 40%–46%, the insulin dose remained considerably low^[36,38]^. Moreover, similar to the current case, anti-GAD levels decreased significantly within the subsequent 24 months post-HSCT^[37]^. However, interpretation is limited by the insufficient duration of follow-up regarding glycemic control in HSCT-treated T1DM patients^[39]^.

Another explanation for improved glycemic control post-HSCT may be that stable donor chimerism following second HSCT may have reduced infections and inflammation. It is known that the bone marrow regulatory network becomes chronically disrupted during diabetes, contributing to immune dysregulation, impairing host defense, and increasing susceptibility to infection^[40]^. In support of this, recent findings indicate that bone marrow replacement can improve the disrupted environment, reducing inflammatory markers and improving glycemic homeostasis in a non-obese, genetically derived animal model for T2DM (Goto-Kakizaki rats)^[41]^. Given that calcineurin inhibitors or agents used for conditioning regimen (busulphan, treosulphan, fludarabine, anti-thymocyte globulin) may theoretically attenuate pancreas inflammation, their potential association with improved glycemic control following HSCT should also be considered. Earlier studies on the effects of CsA demonstrated that its use in new onset T1DM results in preservation of pancreatic *β*-cell function for less than 12 months, while its adverse effects limited its clinical application^[42,43]^. However, in the current case, CsA was used in an established diabetes with near complete loss of *β*-cell function as determined by insulin requirement. Furthermore, the reduction in total daily insulin dose was maintained even after cessation of CsA. The effect of mycophenolate mofetil was also investigated in previous research, which showed that it fails to preserve pancreatic *β*-cell function as mono- or combined treatment in patients with new-onset T1DM^[44]^. These medications are studied to research preservation of *β*-cells; thus, there is no study analyzing effects in established T1DM.

The impact of HSCT on celiac disease has also been previously examined. Ciccocioppo et al. reported two patients who recovered from celiac disease following HSCT with a myeloablative conditioning regimen for * β *-thalassemia major. Despite gluten intake, all clinical, serological, and histological features of celiac disease in their cases remained negative for up to five years^[45]^.

Limitations of this study must be acknowledged. First, as this is a case report, the absence of a comparative cohort limits the generalizability and causal inference of our findings. To substantiate our hypothesis, additional cases and randomized controlled trials are required. However, in rare forms of IEIs, characterized by significant heterogeneity, reports of single cases serve as the starting point for data collection.

## Conclusions

This is the first case of homozygous missense mutation of *CD70*, causing CD70 deficiency presenting with severe onset T1DM in humans. Timely management with HSCT was curative for CD70 deficiency and reduced the total daily dose of insulin with improved glycemic control. This case highlights the significance of intact CD27/CD70 co-stimulation for the prevention of pancreatic *β*-cell autoimmunity. The CD27/CD70 pathway may represent a potential target for future immunotherapy trials in T1DM.

## Data Availability

All data generated or analyzed during this study are included in this published article.
